# Technical Note: Extended field‐of‐view (FOV) MRI distortion determination through multi‐positional phantom imaging

**DOI:** 10.1002/acm2.13065

**Published:** 2020-10-19

**Authors:** Emil Schüler, Richard Mallozzi, Joshua Levy, Dimitre Hristov

**Affiliations:** ^1^ Department of Radiation Oncology Stanford School of Medicine Stanford University Stanford CA USA; ^2^ The Phantom Laboratory Inc Salem NY USA

**Keywords:** compact phantom, distortion map, geometric distortion, image fusion, MR‐linac

## Abstract

Comprehensive characterization of geometric distortions for MRI simulators and MRI‐guided treatment delivery systems is typically performed with large phantoms that are costly and unwieldy to handle. Here we propose an easily implementable methodology for MR distortion determination of the entire imaging space of the scanner through the use of a compact commercially available distortion phantom. The MagphanRT phantom was scanned at several locations within a MR scanner. From each scan, an approximate location of the phantom was determined from a subset of the fiducial spheres. The fiducial displacements were determined, and a displacement field was fitted to the displacement data using the entire multi‐scan data set. An orthogonal polynomial expansion fitting function was used that had been augmented to include independent rigid‐body transformations for each scan. The rigid‐body portions of the displacement field were thereafter discarded, and the resultant fit then represented the distortion field. Multi‐positional scans of the phantom were used successfully to determine the distortion field with extended coverage. A single scan of the phantom covered 20 cm in its smallest dimension. By stitching together overlapping scans we extended the distortion measurements to 30 cm. No information about the absolute location or orientation of each scan was required. The method, termed the Multi‐Scan Expansion (MSE) method, can be easily applied for larger field‐of‐views (FOVs) by using a combination of larger phantom displacements and more scans. The implementation of the MSE method allows for distortion determination beyond the physical limitations of the phantom. The method is scalable to the user’s needs and does not require any specialized equipment. This approach could open up for easier determination of the distortion magnitude at distances further from the scanner’s isocenter. This is especially important in the newly proposed methodologies of MR‐only simulation in RT and in adaptive replanning in MR linac systems.

## 1 INTRODUCTION

MR images are increasingly used in radiation therapy (RT) due to their superior soft tissue contrast allowing for increased accuracy in tumor and normal tissue delineation as compared to segmentation performed on CT images.^1^, ^2^, ^3^, ^4^, ^5^ The preferred method of incorporating the MRI into the clinical workflow has been through rigid registration to a CT image set. The MR‐defined contours are thus mapped onto the CT and the CT is then used for dose calculations and evaluation. Current research is focused on the use of MR simulation alone without the use of an underlying CT scan for dose calculation.^6^, ^7^, ^8^ This would negate the need for multiple scans, thus relieving stress on the patient and eliminating the CT imaging dose. This would also open up the field of adaptive replanning in integrated MR/linac systems.^9^, ^10^

However, a drawback with MRI is the inherent geometric image distortion due to imperfections in the hardware of the system (system dependent geometric distortion) or due to distortions induced by the patient (patient dependent geometric distortion). Patient dependent distortion arises from magnetic susceptibility and chemical shift effects and is more difficult to correct for than system dependent distortion. For this reason, the distortion correction efforts have mostly been concentrated on the latter. Patient dependent distortion is generally also smaller in magnitude.^1^

System distortion arises due to inhomogeneities in the main magnetic field and due to nonlinearities in the induced gradient fields.^3^ As such, the system distortion will be dependent on the pulse sequence type and imaging parameters used, with increased distortion as a function of distance from the isocenter and strength of the main magnetic field.^1^, ^2^ To account for this, vendors of MRI systems supply the user with distortion correction algorithms. However, despite vendor provided 3D distortion corrections, maximum distortion values of 2–3 mm are still routinely measured on these systems.^1^ The clinical effect of this residual distortion is highly site and technique dependent. The consensus of leading experts considers a system distortion of <1 mm in the Stereotactic Radiosurgery (SRS) setting and <2 mm elsewhere acceptable.^1^, ^4^, ^11^ However, a <1 mm residual distortion should also be considered in non‐SRS settings to limit the detrimental effect on plan quality indices to below 5%.^1^, ^5^

Another issue arises with MR‐only simulation in the context of stereotactic body radiation therapy (SBRT) treatments. During these treatments the use of non‐coplanar beams are often utilized with beams entering and exiting the patients at much greater distances from the isocenter than what is seen in conventional treatments. With increased distance from the isocenter the distortion increases and therefore a need for accurate knowledge about the distortion at these off‐target locations is essential to properly account for and protect organs‐at‐risk.

Phantoms are commercially available to accurately quantify MRI distortions. One of the challenges that remains, however, is measuring distortion over the entire MR bore in a practical way. Liquid‐filled phantoms large enough to cover torso‐sized regions of the bore are heavy to handle and unwieldy in a clinical environment. Nonliquid‐filled phantoms can cover a larger region, but are often still unwieldy due to their weight, and perform only distortion measurements. It is desirable to have a phantom that is small enough to be handled easily in a clinical environment and can perform a multitude of measurements so that an overall quality control protocol is simplified.

Here we propose an easily executed methodology for enlarging the dimensions of the MR distortion map through the use of a compact commercially available distortion phantom. By importing several off‐center locations into the distortion calculation algorithm, we can effectively emulate a whole‐bore phantom for accurate, reproducible, and practical determination of distortion beyond the physical volume of the phantom and facilitate the increased use of MRI in radiation therapy.

## 2 MATERIALS AND METHODS

### 2.A Phantom and scanners

**Fig. 1:**
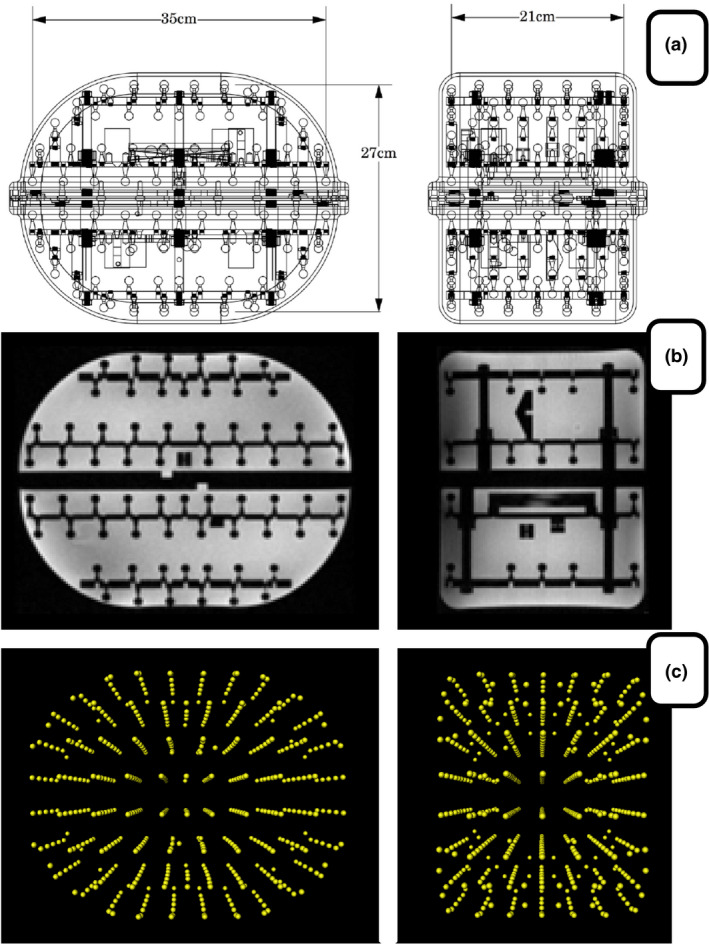
(a) Schematic illustration of the MagPhanRT phantom. field‐of‐view of the distortion sampling points measures 35 cm (width), 27 cm (height), and 21 cm (length), (b) Axial and sagittal MR image of the phantom, and (c) Three‐dimensional representation of the sphere fiducial positions within the phantom used for distortion evaluation.

The MagPhanRT phantom (The Phantom Laboratory Inc, Salem, NY) was used in this study (Fig. 1).^12^ The distortion component of the phantom consists of 505 solid plastic spheres of diameters 1 and 1.5 cm, surrounded by a background fluid with relaxation time approximately 350 ms at 1.5 Tesla.^12^ The spheres appear dark in an MR image. The spheres within the phantom used for distortion mapping are placed with typical spacing of 3–4 cm. They do not strictly adhere to a regular lattice or pattern. However, this spacing is adequate to characterize the distortion field as distortions are generally slowly varying except near the very extremes of the field‐of‐view (FOV) of the scanner.^11^, ^13^, ^14^ Because the phantom is a liquid‐filled phantom, it can also perform other important measurements needed in a quality assurance protocol such as slice thickness, resolution, SNR, and uniformity. The multi‐purpose capability is of practical importance in clinical environments because an efficient quality assurance protocol is desired due to the frequency of quality assurance tests, which are often done on a daily or weekly basis.

The phantom [Fig. 1(a)] is designed with relatively large spatial coverage in two directions (35 × 27 cm), and smaller coverage in the third dimension (21 cm). For a phantom oriented with the larger dimensions within the slice plane, this asymmetry in dimensions enables larger in‐plane coverage for a single scan of the phantom but reduces the coverage in the slice direction. This choice makes the phantom more practical for scans where a large coverage in the slice direction is not necessary. It also simplifies workflow for acquiring multiple scans to extend the coverage, as the table of the scanner can be translated without any manual repositioning of the phantom and coils on the patient table.

The phantom was scanned in a Siemens 3T Skyra Magnetom MRI scanner (Siemens Medical Solutions, Erlangen, Germany) using the body coil and a gradient echo 3D sequence (TurboFLASH) with TE, TR, and flip angle of 2.44 ms, 2200 ms, and 13 degrees, respectively. The pixel size was 1.5625 × 1.5625 mm^2^ with a slice thickness of 1 mm. The scanning FOV was chosen to encompass the entire phantom (380 x 300 x 240 mm^3^ with 240 slices in the Superior/Inferior (Sup/Inf) direction). The 3D vendor supplied distortion correction was applied to all scans. A Siemens Biograph mCT 128 slice CT scanner (Siemens, Muenchen, Germany) was used for internal sphere position verification (120 kVp, 350 mAs, 0.875 × 0.875 × 0.75 mm^3^ voxel size, reconstructed FOV (380 × 300 × 240 mm^3^ with 320 slices in the Sup/Inf direction). The geometric distortion of the CT scanner was evaluated with the CatPhan phantom (The Phantom Laboratory, Salem, NY) and was determined to be 0.1 mm using the vendor recommended protocol.

The phantom was placed on the table such that the dimensions of larger coverage lay in the axial plane. The FOV was extended in the third direction with two additional scans, one with the patient table translated five centimeters in the Sup direction, and one with the patient table translated five centimeters in the Inf direction.

### 2.B Extended FOV distortion determination

**Fig. 2:**
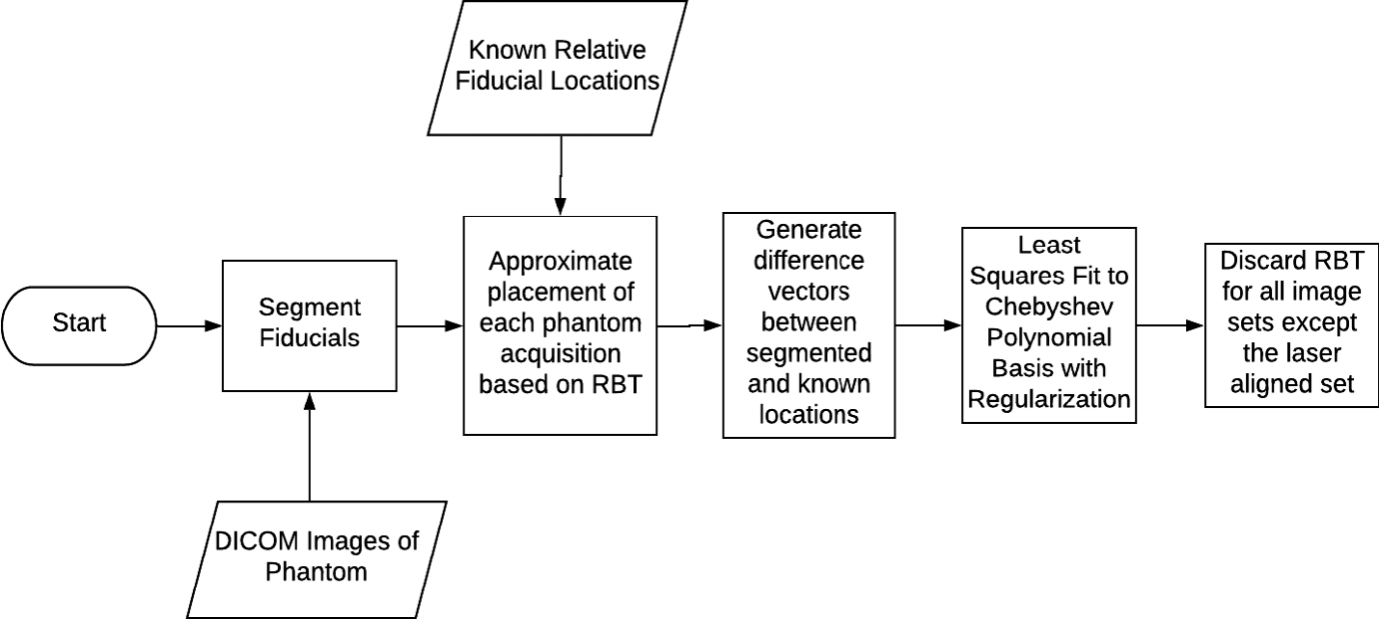
Flow chart of the Multi‐Scan Expansion (MSE) methodology for extended field‐of‐view distortion determination using a standard distortion phantom. DICOM images of the phantom scanned at multiple positions within the scanner are acquired and processed using Matlab. The fiducial spheres are first segmented. Based on a subset of fiducials, a rigid‐body transformation (RBT) is applied to each phantom scan to determine the phantom placement within the scanner. A displacement vector is calculated for each fiducial and the resultant displacement field is fit to a polynomial expansion. From the central phantom position, the misalignment between the laser alignment and the MR coordinate system is determined and applied. The rigid‐body transformations associated with each of the phantom acquisitions are discarded from the resulting fit.

A workflow chart of the Multi‐Scan Expansion (MSE) methodology of going from a single scan limited FOV (LFOV) to a multi‐scan extended FOV (EFOV) for distortion determination is presented in Fig. 2. The LFOV scan was always performed with the phantom in the center of the scanner as determined by the alignment system. A detailed description of the methodology is as follows:

#### 2.B.1 Segmentation

All calculations were performed with custom code written in Matlab, Rev 2016b (MathWorks, Inc, Natick, MA). The fiducial spheres are segmented using Normalized Cross Correlation (NCC). The template for the NCC calculation is generated mathematically from the known dimension of the sphere on a grid. The grid density is five times higher than the voxel density in each of the three dimensions, for a total oversampling factor of 125. The final template pixels are generated by volume averaging at the original voxel size followed by down sampling to generate a template with voxel spacing matching that of the original image. This oversampling‐averaging gives the template sensitivity to partial‐volume effects from the finite voxel resolution. After locating the spheres approximately with NCC, the NCC function is interpolated by a factor of eight in each direction using sinc interpolation to provide sub‐voxel resolution on the sphere location.

The segmented fiducial location determined within the image will be referred to as the ‘segmented’ location. The manufacturing tolerance of the sphere locations is approximately 0.5 mm. A CT scan of the phantom is performed to further increase the accuracy of the known locations, segmented with the same NCC technique, bringing the uncertainty down to approximately 0.2–0.3 mm for typical voxel dimensions. These locations then represent the best‐known position of the spheres, but in an arbitrary coordinate system different from that of the MR scanner. This coordinate system will be referred to as the ‘phantom’ coordinate system. A rigid‐body transformation (three translational and three rotational degrees of freedom) will be needed to know the actual location of the fiducials in the scanner. The means to obtain this transformation are described subsequently.

#### 2.B.2 Localization

An approximate location and orientation of each phantom module within the scanner is determined by first locating three spheres of slightly larger diameter (1.5 cm) than the rest of the fiducial spheres. The spheres are located near the central portion of the phantom, arranged at the vertices of a scalene triangle so that they can be uniquely identified. The Orthogonal Procrustes algorithm is then used to generate a rigid‐body transformation that best transforms the locations of the three spheres in the phantom coordinate system to their locations in the coordinate system of the MR scanner. Because the phantom is itself a rigid body, this transformation can then be applied to any sphere in the phantom to transform its coordinates from the phantom coordinate system to the coordinate system of the MR scanner. The location of each fiducial sphere arrived at in this manner will be referred to as the ‘designed’ location. Because only three spheres are used, and because the sphere locations are subject to geometric distortion in the scanner, the rigid‐body transformation computed thus far (and hence the ‘designed’ location) is still approximate. The residual error is in the form of a small rigid‐body transformation that, as explained below, will be determined from the full distortion fit and does not propagate to the distortion measurements.

#### 2.B.3 Displacement field

From the segmented locations of each sphere determined from the MR image, a displacement vector at each location is calculated as the difference between the segmented location and the designed location. The displacements are fit to an expansion of Chebyshev polynomials:

(1) $$\vec{d}=\Sigma_{m,n,p,i}A_{\mathrm{mnpi}}\Phi_{\mathrm{mnp}}^{\left(i\right)}$$

where the basis functions $\Phi_{\text{mnp}}^{\left(i\right)}$ are

$$\Phi_{\text{mnp}}^{\left(1\right)}=\left(\begin{array}{c} U_{m}\left(x\right)U_{n}\left(y\right)U_{p}\left(z\right)\\ 0\\ 0 \end{array}\right),\;\Phi_{\mathrm{mnp}}^{\left(2\right)}=\left(\begin{array}{c} 0\\ U_{m}\left(x\right)U_{n}\left(y\right)U_{p}\left(z\right)\\ 0 \end{array}\right),\;\Phi_{\mathrm{mnp}}^{\left(3\right)}=\left(\begin{array}{c} 0\\ 0\\ U_{m}\left(x\right)U_{n}\left(y\right)U_{p}\left(z\right) \end{array}\right)$$

In these basis functions, $U_{m}$ is a Chebyshev polynomial of the second kind of order m, and $A_{\mathrm{mnpi}}$ is the fitting coefficient to be determined for the $\Phi_{\text{mnp}}^{\left(i\right)}$ basis function. The choice of polynomial basis is not critical for performing such a fit, as polynomial bases can be constructed from one another with simple linear combinations.

The appropriate order of the fit in Eq. (1) is governed by the structure and orientation of the phantom. The phantom used in this study has internal fiducials that are approximately on a three‐dimensional grid. The order of the fit in each direction [the maximum values of m, n, p in Eq. (1)] is then taken as the number of fiducials along each direction, with a limit placed on the sum m + n + p of 1.5 times larger than the highest of the three integers m, n, and p. This choice is not critical. The aim is to have more than enough terms initially to fit the distortion field, and then apply Tikhonov Regularization to reduce the effective order of the fit to prevent overfitting.

Among the basic functions described above are terms with no spatial dependence (m = 0, n = 0, p = 0), which represent simple translations by a distance δ along the x, y, or z axes:

$$T_{1}=\delta \left(\begin{array}{c} 1\\ 0\\ 0 \end{array}\right),\;T_{2}=\delta \left(\begin{array}{c} 0\\ 1\\ 0 \end{array}\right),\;T_{3}=\delta \left(\begin{array}{c} 0\\ 0\\ 1 \end{array}\right)$$

Also among the basis function set described above are functions where each component is proportional to only x, y, or z, where either m, n, or p is 1 and the other two are zero. The subspace spanned by these components can be described in linear combination of these vectors that include terms to represent distortions corresponding to small, linearized rotations and translations. Small rotations about the x, y, and z axes, respectively, are represented by the following distortion basis functions:

$$R_{x}\left(x,y,z\right)=\left(\begin{array}{c} 0\\ ‐z\\ y \end{array}\right),\;R_{y}\left(x,y,z\right)=\left(\begin{array}{c} z\\ 0\\ ‐x \end{array}\right),\;R_{z}\left(x,y,z\right)=\left(\begin{array}{c} ‐y\\ x\\ 0 \end{array}\right)$$

Thus, by simple rearrangement of the basis functions described in Eq. (1), the translational and small rotational components can be broken out as follows:

(2) $$\vec{d}\left(x,y,z\right)=\sum_{j=1}^{j=3}\delta_{j}T_{j}+\sum_{j=1}^{j=3}\epsilon_{j}R_{j}\left(x,y,z\right)+\sum_{\mathrm{mnpi}}^{\prime }A_{\mathrm{mnpi}}\Phi_{\mathrm{mnp}}^{\left(i\right)}\left(x,y,z\right)$$

where the prime over the last summation term in Eq. (2) refers to the exclusion of the six bascs functions from Eq. (1) that were incorporated into the first two sums.

Equation (2) is an expression for the displacement field within the MR scanner to be measured with the phantom. The displacement vector $\vec{d}\left(x,y,z\right)$ represents the difference between the apparent location of the point that appears in the image at location $\left(x,y,z\right)$ and the designed location. The form of Eq. (2) has a simple physical interpretation. The first summation containing basis vectors $T_{j}$ represents a rigid‐body translation of all the fiducials (i.e. the entire phantom). The second term with the $R_{j}$ basis functions represents a small rigid‐body rotation of all fiducials about the origin of the scanner coordinate system. The remaining terms represent geometric distortions of the image, rather than translations or rotations.

Because of the approximate rigid‐body transformation performed using the Procrustes Algorithm on the three larger spheres, the translational and rotational terms in Eq. (2) will be small and represent corrections to that original transformation. These terms can be used to perform such a correction to that transformation and thereby identify the actual location and orientation of the overall phantom location within the scanner. This information is highly valuable in assessing mismatch between the laser alignment system of the MR scanner and the actual coordinate system of the MR scanner. Such mismatch can occur not only from a mechanical misalignment of the laser system and the MRI gradient system, but also from a center frequency offset error, which introduces translations in the MR image. The final apparent position of the phantom in the scanner is an output of the distortion fit that contains information that can be conceptualized equivalently as either a rigid‐body distortion, or as a misalignment between the laser alignment system and the MRI coordinate system.

When combining multiple image series to extend the FOV, distinct translational and rotational terms need to be applied for each separate image series to account for the different phantom placements for each series. The distortion terms (with the $\Phi_{\mathrm{mnp}}$ basis functions) are common to all image series, as they are a property of the scanner, not the placement of the phantom. We can thus extend Eq. (2) to deal with multi‐scan acquisitions by assigning the index *s* to label the different series. Defining translational and rotational basis functions $T_{j}^{\left(s\right)}$ and $R_{j}^{\left(s\right)}$ that operate only on the fiducials from series *s* (leaving fiducials from other series unchanged) will yield.

(3) $$\vec{d}\left(x,y,z\right)=\sum_{s,j}\delta_{s,j}T_{j}^{\left(s\right)}+\sum_{s,j}\epsilon_{s,j}R_{j}^{\left(s\right)}\left(x,y,z\right)+\sum_{\mathrm{mnpi}}^{\prime }A_{\mathrm{mnpi}}\Phi_{\mathrm{mnp}}^{\left(i\right)}\left(x,y,z\right)$$

Among the translational and rotational terms in Eq. (3), the only ones that contain meaningful information are those associated with the series for which the phantom was placed at isocenter and aligned with the laser alignment system. This information can be used to detect translational and rotational mismatch of the alignment system with the MR coordinate system as described earlier. The other translational and rotational terms, associated with the translated image series, contain information only about the somewhat arbitrary translations applied to those series, rather than information about the geometric distortion of the scanner. The geometrical distortion information is all contained in the final summation term (with the $\Phi_{\mathrm{mnp}}^{\left(i\right)}$ basis functions) in Eq. (3). Discarding the translational and rotational terms amounts to using only the relative locations of the fiducials for the additional phantom acquisitions and renders knowledge of the absolute location of each phantom placement unnecessary.

The procedure is as follows:

1. Acquire multiple MR image series of the phantom, with the phantom placed in different locations, with substantial degree of overlap in coverage region. To get the translational and rotational information about the alignment system, one of the phantom acquisitions should be performed at the center of the device as determined by the alignment system.
2. Segment the fiducials from each series to get an absolute (apparent) location of each fiducial marker
3. For each series, determine an approximate location of each phantom by any convenient method, such as using a small number of landmark fiducials that can be segmented easily.
4. Based on the approximate location of the phantom, use the known design of the phantom fiducials and the measured location within the images to determine a displacement measurement $\vec{d_{k}}$ for each fiducial *k*, which is found within the image at location $\left(x_{k},y_{k},z_{k}\right)$.
5. Perform the fit described in Eq. (3) to the collection of fiducial locations.
6. The translational and rotational terms in Eq. (3) for the series where the phantom was placed at center determine the translational and rotational misalignment between the MR system and the alignment system
7. The translational and rotational components of all other series can be discarded, and the true distortion field of the MR scanner determined by the terms in the last summation in Eq. (3).

In applying the fit with Eq. (3), Tikhonov Regularization is used to prevent overfitting of the distortion field.

It is useful to understand the impact of center frequency on such distortion measurements. The center frequency is generally calibrated immediately prior to a set of acquisitions, with the actual patient or phantom in the scanner. Errors in the center frequency calibration manifest themselves as translations of the entire image in the readout direction, with a magnitude inversely proportional to the readout bandwidth. Generally, such translational errors will amount to a small fraction of a pixel dimension. In this technique, it is not important to recalibrate center frequency between phantom placements, as the translational and rotational information for all but the center placement is discarded. Any errors in the center frequency calibration will be reflected in the translational measurement of the displacements that comes out of the fit in Eq. (3).

### 2.C Method evaluation and validation

To evaluate the distortion correction accuracy, a high‐quality CT of the phantom was acquired. This CT image was used as our “gold standard” for determining the sphere fiducial locations within the phantom. The corrected MR images were compared against the CT image and the overall absolute difference in sphere fiducial position and the dependence of absolute difference in sphere position on distance from the MR isocenter was determined.

A further evaluation of the distortion correction was performed based on the internal distances between sphere positions within the individual scans. In this evaluation, the EFOV vendor‐corrected MR images and the CT images were used directly. The EFOV vendor‐corrected images refer here to the use of the multiple translated sets of MR images, each of which has had the vendor’s standard built‐in distortion correction applied. The EFOV vendor‐corrected MR images were also further corrected based on the distortion determined through the MSE method. These images are hereafter termed MSE corrected MR images. The relative distances between the sphere positions within the two MR image sets were compared with that determined from the CT images. This eliminated the need to define the location of the phantom in any of the scans. The absolute difference in distance between each sphere between the MR image sets and the CT was calculated for all different sphere combinations (number of distances compared = n(n + 1)/2 − n (n = number of spheres)).

## 3 RESULTS

### 3.A Distortion determination

**Fig. 3:**
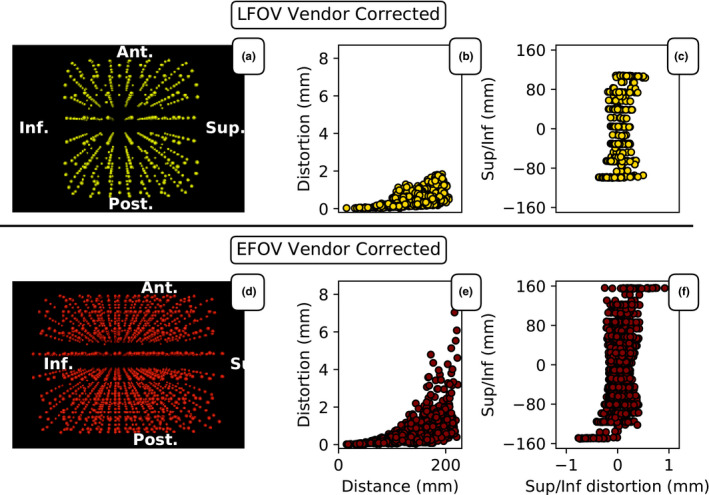
(a) and (d) three‐dimensional representation of the sampling points within the phantom used for distortion determination for limited field‐of‐view (LFOV) and extended FOV, respectively. (b) and (c) shows the distortion magnitude as a function of distance from the MRI isocenter and the Sup/Inf distortion in the Sup/Inf direction, respectively, measured with LFOV (single phantom position). Corresponding plots for EFOV (multiple phantom positions) are shown in (e) and (f). In the EFOV, the phantom was translated in the Sup/Inf direction by ±5 cm, thereby extending the distortion map dimensions.

The number of fiducial locations was increased from 505 to 1515 positions through the inclusion of a ±5 cm translation in the Sup/Inf direction [Fig. 3(a) and 3(d)]. The increase in sampling points density and the extended FOV is seen in Fig. 3(d). The maximum distortion determined with LFOV was 1.8 mm [Fig. 3(b)]. The maximum distortion determined with EFOV was 7.0 mm [Fig. 3(e)]. The average distortion increased with increased distance from the isocenter [Figs. 3(c) and 3(f)].

**Fig. 4:**
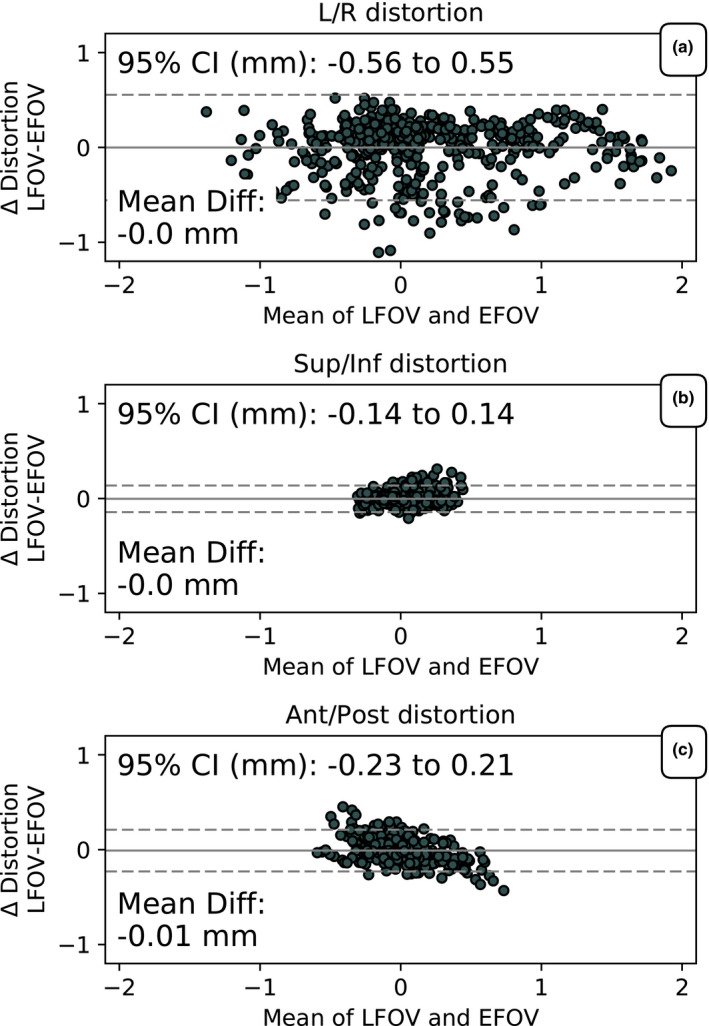
The difference in distortion vs the mean distortion of limited field‐of‐view (LFOV) and extended FOV. The mean difference is indicated by the solid line and the 95% CI is indicated by the dotted lines. The distortion was divided into (a) L/R, (b) Sup/Inf, and (c) Ant/Post directions.

The differences in the distortion maps determined through LFOV and EFOV were evaluated by comparing the distortion at the sphere positions from the central phantom position in the EFOV data set to the LFOV data set (Fig. 4). The physical positions of the spheres between these two data sets are the same and any difference is thus only due to differences in the fit of the fiducial locations. The mean distortion between the two approaches was the same. The average corrected sphere position difference between the two approaches was determined to be 0.3 ± 0.2 mm. Two out of the 505 spheres had a positional difference of >1 mm (1.1 mm difference). The largest difference in the determined distortion was in the Left/Right (L/R) direction where the 95% confidence interval (CI) was −0.56 to 0.55 mm. The differences in the determined distortion in the Sup/Inf or in the anterior/posterior (Ant/Post) direction were more than a factor of 2 smaller.

### 3.B Method evaluation and validation

**Fig. 5:**
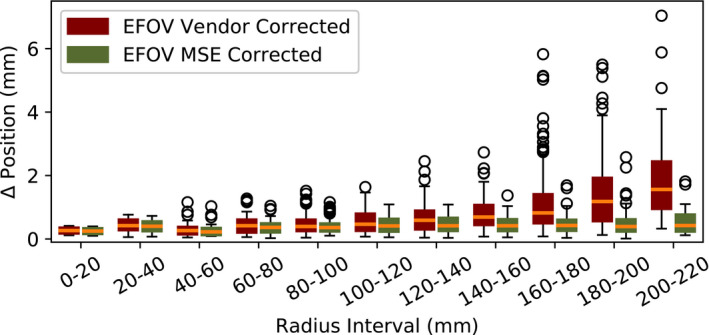
The dependence of absolute sphere position disagreement on radial distance from the MR isocenter for extended field‐of‐view (EFOV) vendor‐corrected MR and EFOV Multi‐Scan Expansion (MSE) corrected MR as compared to CT. The boxes represent the interquartile range (IQR) between the third (Q3) and the first (Q1) quartile. The upper and lower whiskers extend to Q3 + 1.5 * IQR and Q1 – 1.5 * IQR, respectively. The outliers (black circles) are defined as data points that fall below Q1 – 1.5 * IQR or above Q3 + 1.5 * IQR. The orange lines are the median value. The median positional disagreement increased with increased distance from the MR scanner isocenter for the vendor‐corrected MR images. No significant change in positional disagreement with distance from the MR isocenter was found for the MSE corrected MR images.

**Fig. 6:**
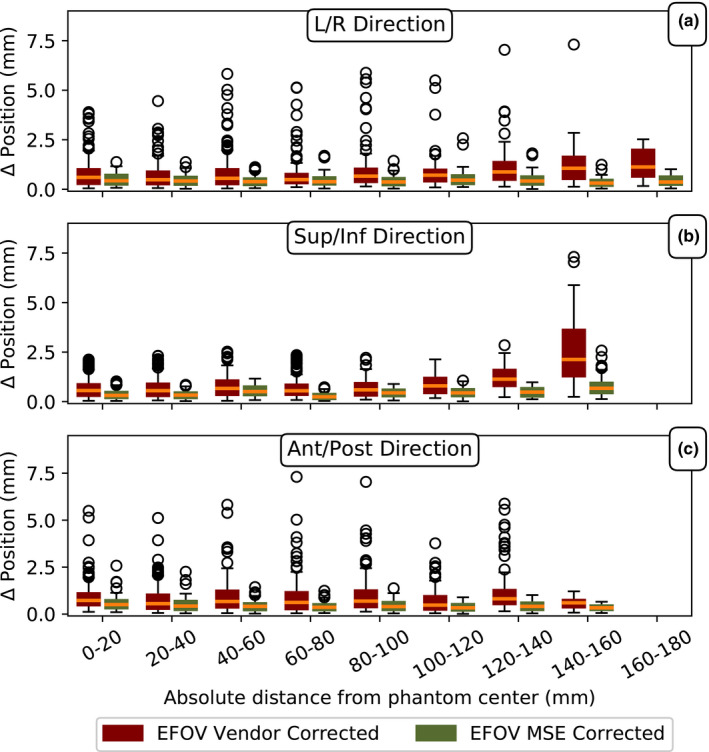
The dependence of absolute sphere position disagreement on (a) L/R, (b) Sup/Inf, and (c) Ant/Post distance from the MR isocenter for extended field‐of‐view (EFOV) vendor‐corrected MR and EFOV Multi‐Scan Expansion (MSE) corrected MR as compared to CT. The boxes represent the interquartile range (IQR) between the third (Q3) and the first (Q1) quartile. The upper and lower whiskers extend to Q3 + 1.5 * IQR and Q1 – 1.5 * IQR, respectively. The outliers (black circles) are defined as data points that fall below Q1 – 1.5 * IQR or above Q3 + 1.5 * IQR. The orange lines are the median value. The median positional disagreement increased with increased distance from the MR scanner isocenter for the vendor‐corrected MR images. No significant change in positional disagreement with distance from the MR isocenter was found for the MSE corrected MR images.

Figures 5 and 6 illustrate the absolute sphere position disagreement between the EFOV MR images and the CT image. The MR images were either corrected for distortion through the vendor supplied algorithm or the MSE algorithm. The dependence of positional disagreement on the radial and the L/R, Sup/Inf, and Ant/Post distance from the MR isocenter is shown in Figs. 5 and 6, respectively. The vendor‐corrected MR image showed increased positional disagreement from the CT determined position with increasing distance from the isocenter of the MR scanner in all directions. The largest increase with distance was in the Sup/Inf direction. The median distortion at 200–220 mm radial distance from the isocenter was 1.6 mm with a maximum positional disagreement of 7.0 mm (Fig. 5). For the MSE corrected MR image, the median positional disagreement from the CT determined position was constant with distance from the isocenter. The median and the maximum positional disagreement at 200–220 mm radial distance from the MR isocenter was 0.4 and 1.8 mm, respectively.

**Fig. 7:**
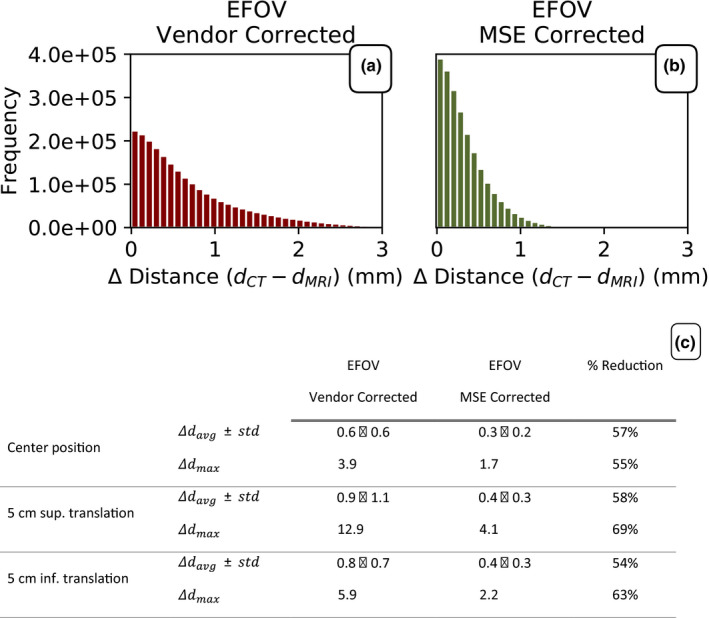
The difference in distance between each sphere in the phantom as determined through segmentation of (a) the vendor‐corrected MR and (b) the Multi‐Scan Expansion (MSE) corrected MR. Both image sets were compared to the segmentation of the sphere position in the computed tomography (CT) image. (c) Average and maximum distance disagreement between the vendor‐corrected/ MSE corrected MR images and the CT image for the three different positions of the phantom in the MR scanner. The percent reduction in Δ distance with the MSE correction was independent of the translational position of the phantom.

To further validate the MSE method and to remove any assumption about the absolute position of the phantom, the internal distances between all fiducial spheres were determined for each image set for each phantom position (Fig. 7). Again, the CT image was considered the golden standard and the distances between the spheres measured within the CT scan were considered the true distances. The MR images acquired with the EFOV with either vendor correction or MSE correction applied were compared to the CT image. The frequency distribution of the difference in distances measured can be seen in Figs. 7(a) and 7(b) for the vendor‐corrected and the MSE‐corrected MR, respectively. A sub‐analysis of the individual phantom positions revealed an average distance disagreement of 0.6, 0.9, and 0.8 mm for the central, +5cm translation, and −5 cm translation in the Sup/Inf direction, respectively, for the vendor‐corrected MR images. The corresponding numbers for the MSE corrected MR images were 0.3, 0.4, and 0.4 mm, respectively. No significant difference in distance disagreement reduction was found between different phantom positions [Fig. 7(c)].

## 4 DISCUSSION

In this study we present an easily executed methodology for extending the FOV of the determined MR distortion map beyond the physical boundaries of a single phantom. The methodology increases the number of distortion sampling points, the FOV, and the resolution of the distortion sampling. This is achieved through image acquisition of a standard distortion phantom in different positions within the scanner. In this study, we used a ±5 cm translation in the Sup/Inf direction to demonstrate our methodology. However, conceptually the methodology is not limited to translational offsets, nor is it limited to inclusion of only three scans, but can instead be scaled to the user’s requirement. The implementation is also independent of the direction of FOV extension. Because no information about the absolute position or orientation of each module is assumed, modules from different scans can be easily combined to enlarge the FOV of the distortion measurement.

The analysis of the distortion magnitude determined with LFOV and EFOV showed a mean difference in sphere position of 0.3 ± 0.2 mm for the overlapping sphere positions. This is within the uncertainty of the segmentation (0.2–0.3 mm uncertainty, based on repeated inter‐sphere distance measurements made with multiple scans of the phantom in slightly displaced locations). This demonstrates that the distortion fits in the overlap region for the two approaches are equivalent. We further demonstrate that the application of the MSE methodology for distortion correction allows for a twofold reduction of the residual mean geometric errors (as measured by inter‐sphere distances).

The standard approach for determining the distortion map for the entire FOV of an MR scanner is through large phantoms that fill the entire bore. While these phantoms do allow for direct determination of the distortion map, the size and reported weights of up to 60 kg makes them practically hard to handle.^11^, ^13^ Furthermore, the availability of these phantoms are mostly limited to research settings.^11^, ^13^ However, one system consisting of layers of light‐weight polyurethane foam material embedded with ellipsoidal markers have been reported with a FOV of 50 × 50 × 50 cm^3^ and a total weight of 5 kg, overcoming some of the limitations with these systems.^14^ While a direct comparison between studies using entire‐bore phantoms and this one would require the same scanning setup and protocol, a comparison of the overall magnitude of the residual distortion between using an entire‐bore phantom and using the MSE methodology revealed similar results.^11^, ^13^, ^14^

Although this multi‐scan approach requires several acquisitions of the phantom, each taking several minutes, there are substantial practical benefits of enhancing the ability of a smaller phantom to cover larger FOV. Routine quality assurance protocols are often performed daily or weekly, and don’t necessarily require large FOV for the distortion measurement. A smaller phantom that can be used to cover the larger FOV on a less frequent basis has clinical value in keeping the primary quality control protocol efficient to execute, and prevents the need for an entirely different phantom to cover large fields of view.

In our study we used distance measurements in 3D phantom images acquired at different offset from the isocenter as surrogate metrics for evaluating the accuracy of our propose method. An alternative, more direct analysis would involve a comparison between the distortion map determined through the proposed methodology and a distortion map determined through the use of a whole‐bore phantom. Along these lines, the validity of the FOV extension was verified in the region of overlap between the LFOV and EFOV data sets, but no such verification could be performed in the parts that extended beyond this region. Furthermore, only one specific imaging protocol was used for MR image acquisition in the current study. Different imaging protocols can have different distortion patterns. The largest difference between protocols arises from using 2D vs 3D acquisition techniques, particularly if the vendor distortion correction is not activated in the slice direction on a 2D acquisition. The other main source of protocol‐to‐protocol variation depends upon the relative importance of gradient nonlinearity vs static magnetic field inhomogeneity. Distortion arising from gradient nonlinearity will be largely independent of protocol (other than the 2D vs 3D issue), whereas distortion arising from static magnetic field inhomogeneity will be sensitive to readout bandwidth as well as the orientation of the scan plane and readout axis direction. The methodology presented here can be repeated to characterize distortion on a protocol‐specific basis.

## 5 CONCLUSION

In summary, we have presented a methodology that could enable the user to use a standard distortion phantom to extend the mapping of MR distortion beyond the physical dimensions of the phantom. The methodology is easily implementable using compact distortion phantoms readily available at most institutions, does not require any specialized equipment, and is scalable to the user’s needs. This approach could open up for easier determination of the distortion magnitude at distances further from the scanner’s isocenter, which is especially important in newly proposed methodologies of MR‐only simulation in RT and in adaptive re‐planning in MR linac systems.

## CONFLICT OF INTERESTS

Richard Mallozzi and Joshua Levy are employed at The Phantom Laboratory Inc. Emil Schüler and Dimitre Hristov have nothing to disclose.
